# Yttrium Ion Release and Phase Transformation in Yttria-Stabilized Zirconia Under Acidic Conditions: Implications for Dental Implant Durability

**DOI:** 10.3390/ma18143311

**Published:** 2025-07-14

**Authors:** Haochen Zhu, Chao-Ching Chiang, Valentin Craciun, Griffin M. Deane, Fan Ren, Josephine F. Esquivel-Upshaw

**Affiliations:** 1 Department of Chemical Engineering, College of Engineering, University of Florida, Gainesville, FL 32611, USA; zhuhaochen0329@gmail.com (H.Z.);; 2 National Institute for Lasers, Plasma and Radiation Physics, 077125 Magurele-Ilfov, Romania; 3 Department of Restorative Dental Sciences, Division of Prosthodontics, College of Dentistry, University of Florida, Gainesville, FL 32610, USA

**Keywords:** zirconia, yttrium, LTD

## Abstract

The stability of yttria-stabilized zirconia (YSZ) as a dental implant material is highly dependent on its resistance to low-temperature degradation (LTD) and surface dissolution, particularly in acidic oral environments. This study investigates the effects of yttrium ion (Y^3+^) release on the phase stability of zirconia during constant immersion and pH cycling tests, simulating oral conditions. Zirconia disks were immersed in acidic (pH 2), neutral (pH 7), and basic (pH 10) solutions over a 27-day period. Inductively coupled plasma (ICP) analysis revealed significant yttrium ion release during acidic phases, while zirconium ion (Zr^4+^) release remained minimal. X-ray photoelectron spectroscopy (XPS) showed a shift in zirconium 3d binding energies, indicating a transformation from the tetragonal to the monoclinic phase, driven by yttrium leaching. X-ray diffraction (XRD) confirmed this phase change, with the appearance of the monoclinic (111) peak after exposure to acidic conditions. This study concludes that yttrium ion depletion under acidic conditions destabilizes the tetragonal phase, promoting LTD and compromising the material’s long-term performance as a dental implant or restorative material.

## 1. Introduction

A dental implant is a treatment option to replace missing teeth. It is a structure implanted into the oral cavity to mimic the appearance and function of a natural tooth. They act as artificial tooth roots made of titanium or other biocompatible materials, surgically inserted into the jawbone. Over time, the implant fuses with the bone through osseointegration, creating a stable foundation [1,2]. While titanium has been a popular material for dental implants, recent research has found that zirconia implants may become more suitable. Zirconia dental implants are chosen for their aesthetic appeal, biocompatibility, and osseointegration, all of which are comparable or more favorable than titanium-based implants [2,3,4]. In terms of osseointegration, studies have found that zirconia is more biocompatible, as the titanium implant may produce corrosion products around the implant. Zirconia also mimics a natural tooth in that its color is similar. This allows for better aesthetic appeal, as titanium implants are gray [2,5]. Another important feature of zirconia implants is their longevity. Studies have shown that the success rates of zirconia dental implants are comparable to titanium implants, showing high long-term success. Rodriguez et al. found a 92% success rate for zirconia dental implants, and a separate study by Shukla et al. found a 93.3% success rate for zirconia dental implants over five years [6,7,8].

However, one significant drawback associated with zirconia in dentistry is low-temperature degradation (LTD), a phenomenon that can compromise their structural integrity over time [9,10,11,12]. The transformation from the tetragonal to monoclinic phase initiates microcrack formation at the surface, which can propagate deeper into the material, further compromising the implant’s integrity. Wulfman et al. found that one significant issue with LTD is found in relation to measurement techniques and the effects of aging. The issue was the limited thickness of the transformed layer observed after short aging periods, which complicates the interpretation of data obtained from cross-sectional measurements. The collection zone in the measurements may overlap with both tetragonal and monoclinic zirconia, potentially leading to inaccurate readings of the volume fraction of the monoclinic phase [13]. Pereira et al. detected that LTD caused an increase in monoclinic phase content, which indicates phase transformation. This transformation introduces residual stress and can lead to surface roughening and defects [14]. Additionally, Davoodzadeh et al. found that increased yttria content improved transparency in the implant but slightly decreased mechanical hardness, showing that balancing optical and mechanical properties is challenging. While these higher yttria samples resist LTD better, they are less robust compared to lower-yttria-content materials, which tend to offer better mechanical performance but are more susceptible to LTD [15]. To mitigate the effects of LTD, Cho et al. co-doped 3% yttria-stabilized zirconia (3Y-TZP) with niobium oxide and tantalum oxide. The modified zirconia exhibited enhanced strength and resistance to degradation while maintaining favorable osteogenic properties [16]. Kolakarnprasert et al. also found that yttrium content, seen in 5%, 4%, and 3% mol yttria partially-stabilized zirconia samples, had a significant impact on its resistance to LTD. Materials with higher yttrium content, like 5% and 4%, which contained a greater proportion of cubic zirconia, were less susceptible to phase transformation and LTD after hydrothermal aging [17].

pH cycling tests are used in dentistry to simulate the dynamic conditions of the oral environment, particularly the fluctuations in pH caused by dietary acids and the buffering action of saliva [18,19,20]. Ceramics, including zirconia, were previously considered inert materials, but recent findings have shown that they can be susceptible to corrosion, particularly in the oral environment, where pH levels fluctuate due to dietary habits [21]. Esquivel-Upshaw et al. showed that ceramics corroded when exposed to both low- and high-pH environments. Cyclic pH exposure, simulating the oral environment’s alternating conditions, has been shown to cause more surface degradation compared to constant immersion in a single pH solution. In a separate study, Esquivel-Upshaw et al. found that corrosion of ceramic materials can reduce their fracture strength and increase surface roughness, which not only affects the longevity of restorations but also leads to greater plaque accumulation, which can cause damage to adjacent teeth and tissues [22]. The susceptibility of ceramic surfaces to pH changes, resulting in decreased chemical durability, is influenced by their microstructure. Surface degradation from corrosion can also increase the release of ions from ceramics, potentially heightening cytotoxic effects on surrounding tissues [23]. Studies have also shown that yttria-stabilized tetragonal zirconia, in particular, exhibits increased surface roughness after being exposed to acidic environments [24]. Yttria-stabilized zirconia (YSZ) ceramics are known to undergo aging, influenced by both physical and chemical factors. This aging is often accelerated by exposure to various chemical agents, which induces the formation of superficial microcracks on the surface. These microcracks weaken the structural integrity of the material over time, contributing to its degradation [25].

In order to investigate the effects of the stability and degradation of YSZ under varying pH conditions, zirconia discs, fabricated to uniform dimensions, were allocated to three distinct groups for analysis: constant immersion in a singular pH environment, acid-to-base cycling, and base-to-acid cycling over a duration of 27 days. Prior to immersion, the discs underwent a drying process to ensure consistency. Each group was subjected to specific pH buffer solutions designed to mimic the conditions of the oral environment. The samples were incubated in a controlled setting to maintain temperature stability. Characterization techniques, including ICP analysis, XPS, and XRD, were employed to evaluate the phase stability and chemical composition of the zirconia disks throughout the study. The following work aimed to provide insight into the implications of yttrium ion release and pH fluctuations on the long-term performance of zirconia as a dental implant material.

## 2. Materials and Methods

Zirconia disks, each measuring 1 cm in diameter and 5 mm in thickness, were separated into three groups: (1) constant immersion, (2) 3-day acid (pH 2) to base (pH 10) cycling, and (3) 3-day base (pH 10) to acid (pH 2) cycling. Three disks were allocated to each group. Before the experiment, all disks were dried in an oven at 100–105 °C for 24 h, then cooled in a desiccator. The disks were immersed in three buffer solutions: pH 10 (potassium carbonate–potassium borate–potassium hydroxide, SB116-500, Fisher Chemical, Pittsburgh, PA, USA), pH 2 (potassium chloride–hydrochloric acid, SB96-500, Fisher Chemical, Pittsburgh, PA, USA), and pH 7 (potassium phosphate monobasic–sodium hydroxide, SB108-500, Fisher Chemical, Pittsburgh, PA, USA), following specific immersion sequences. For the acid-to-base cycling group, the sequence was pH 2, pH 10, followed by pH 7. Conversely, for the base-to-acid group, the sequence was pH 10, pH 2, followed by pH 7. Each disk was immersed in either pH 2 or pH 10 for the first 3 days, then pH 10 or pH 2 for the next 3 days, and finally in pH 7 for another 3 days, with the entire cycle repeated over 27 days. In contrast, the constant immersion group remained in the same buffer solution throughout the 27 days without cycling. Each set of disks was continuously soaked in pH 2, pH 10, or pH 7, allowing for a more stable exposure to a single pH environment. Following the immersion cycles, all disks were placed in polyethylene centrifuge tubes (Thermo Scientific Nalgene Oak Ridge High-Speed Centrifuge Tubes, Thermo Fisher Scientific, Waltham, MA, USA) and incubated in a shaking water bath (TSBS40, Techne, Vernon Hills, IL, USA) at 80 °C with 50 oscillations per minute.

Zirconium and yttrium ion concentrations released during each phase of the pH cycling were measured using an inductively coupled plasma atomic emission spectrometer (ICP, 3200RL, PerkinElmer, Waltham, MA, USA). Additionally, the surface chemical composition of one disk, which had been immersed in the acidic solution (pH 2) for 27 days, was analyzed by X-ray Photoelectron Spectroscopy (XPS) with an ESCALAB 250Xi instrument (Thermo Fisher Scientific, Pittsburgh, PA, USA) using a monochromatic aluminum X-ray source. High-resolution scans were performed at an electron pass energy of 20 eV and an energy step size of 0.1 eV, specifically targeting the zirconium 3d and yttrium 3d regions. To remove surface contamination, the spectra were acquired after a light 150 s sputtering with an argon cluster beam followed by 500 eV Ar ion sputtering to remove a thin atomic layer from the surface. X-ray diffraction (XRD) analysis was also conducted on the disks from each immersion and cycling condition with a control sample that had not undergone pH immersion, using a MiniFlex XpC (Rigaku, Tokyo, Japan), with generator voltage and tube current of 45 kV and 40 mA and a Cu Kα radiation source (λ = 1.5406 Å) to assess the crystal structure of the sample surface. The scan range was set from 20° to 80° (2θ) with a step size of 0.03°. The exposure time for each step was 2.64 s.

## 3. Results

**Figure 1:**
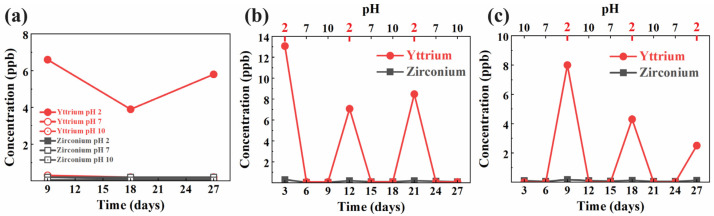
ICP analysis of the zirconium and yttrium content released in solution after different pH cycles: (**a**) in constant pH environment, (**b**) in 2-7-10 sequenced pH environment, and (**c**) in 10-7-2 sequenced pH environment.

The results of the inductively coupled plasma (ICP) analysis, as shown in Figure 1a–c, demonstrated the concentration of yttrium and zirconium ions released over time during the pH cycling test. In Figure 1a, the constant immersion group shows a steady release of yttrium ions in acidic conditions (pH 2), with concentrations ranging between 4 and 6 ppb throughout the 27-day period. In contrast, the release of zirconium remains minimal under all pH conditions (2, 7, and 10), staying below 2 ppb. Figure 1b highlights the acid-to-base cycling group, where significant yttrium ion release peaks are observed at the beginning of each acidic phase (days 3, 9, 15, 21, and 27), reaching concentrations of 12 to 14 ppb, while yttrium release during base phases is negligible. Zirconium levels, on the other hand, remain consistently low throughout the cycle. Similarly, Figure 1c illustrates the base-to-acid cycling group, with yttrium ion release peaking primarily during the acidic phases (pH 2) on days 9, 15, 21, and 27, although the concentrations are slightly lower compared to the acid-to-base cycling group, with a maximum release of around 8 ppb. Once again, zirconium ion release is minimal across all cycles. These results suggest that the acidic environment plays a key role in promoting yttrium ion release from the zirconia disks, while zirconium release remains negligible regardless of the pH conditions.

**Figure 2:**
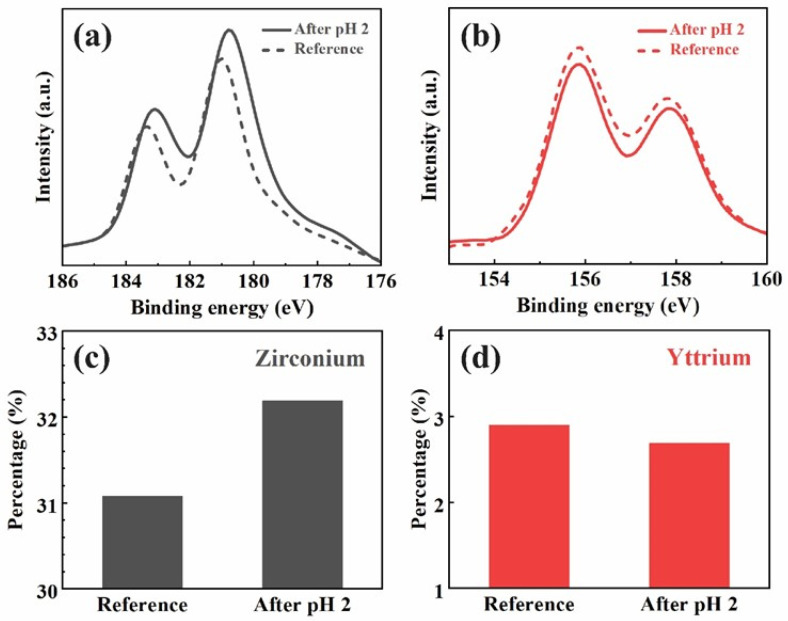
XPS results of the sample immersed in pH 2 solution for 27 days: (**a**) Zr 3d region, (**b**) Y 3d region, (**c**) Zr concentration change after immersion, and (**d**) Y concentration change after immersion.

The XPS analysis presented in Figure 2 shows the binding energy patterns of zirconium and yttrium for the constant acid immersion sample after 27 days, compared to a reference sample. Figure 2a demonstrates the sample after pH 2 immersion with a Zr 3d5/2 peak of 180.7 eV and a Zr 3d3/2 peak of 183.1. Compared with the reference sample of a Zr 3d5/2 peak of 181 eV and a Zr 3d3/2 peak of 183.4, a noticeable −0.3 eV peak shift can be observed in the Zr 3d binding energy after the immersion. Figure 2b illustrates the Y 3d binding energy, where no significant shift in the yttrium peak is observed between the pH 2-immersed sample and the reference. The compositional analysis calculated by the area under the patterns, as shown in Figure 2c,d, reveals an increase in the surface concentration of zirconium from 31.5% in the reference sample to 32.5% in the pH-cycled sample. In contrast, the yttrium surface concentration slightly decreases from 3.0% in the reference sample to 2.8% after pH cycling.

**Figure 3:**
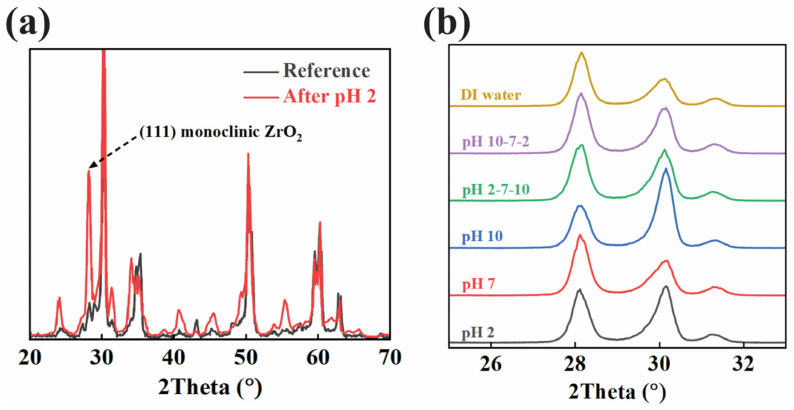
XRD results of the sample immersed in (**a**) pH 2 solution for 27 days and (**b**) all conditions.

The XRD patterns shown in Figure 3a compared the crystal structures of the zirconia disks before and after immersion in pH 2 for 27 days. The reference sample exhibits a typical diffraction pattern consistent with the tetragonal phase of zirconia, marked by peaks at around 30°, 50°, and 60° (2θ). In contrast, the sample exposed to pH 2 shows the emergence of a new peak near 28.2° (2θ), which corresponds to the (111) plane of monoclinic ZrO_2_. This new peak suggests a transformation from the tetragonal to the monoclinic phase in the acid-immersed sample, as indicated by the intensity increase in the monoclinic region. In Figure 3b, more XRD results were recorded and compared with each other. Focusing on the two phases of ZrO2, we can find the same phenomenon of the coexistence of the two phases across all the different immersion conditions.

## 4. Discussion

The stability of zirconia as a dental implant material is highly dependent on its resistance to low-temperature degradation (LTD) and surface dissolution, particularly in the acidic environment of the oral cavity. Yttria-stabilized zirconia (YSZ), a widely used material, incorporates yttrium into the zirconia lattice to maintain the material in its tetragonal phase, thus mitigating LTD. The addition of yttrium creates a tetragonal phase by substituting zirconium ions (Zr^4+^) and generating oxygen vacancies that prevent the transformation to the monoclinic phase, a process known to occur in moist environments and low temperatures. These vacancies enhance the mechanical durability of the zirconia by preventing the volumetric expansion typically associated with phase transformation [26,27,28].

This stabilization mechanism, where yttrium ions (Y^3+^) bond with oxygen atoms (O^2−^) within the zirconia lattice, is essential for maintaining structural integrity. Without this stabilization, zirconia would be prone to surface roughening and microcracking, significantly compromising its mechanical performance in dental applications. In the oral cavity, where implants are exposed to varying pH levels, the choice of 3 mol% yttria-stabilized zirconia (3Y-TZP) optimizes the balance between strength and LTD resistance. However, under acidic conditions, particularly during pH cycling tests designed to simulate oral environments, yttrium ions may be released from the zirconia matrix. This process is initiated by surface protonation from hydrogen ions (H^+^) in the acidic solution, which weakens the bonds between Y^3+^ and O^2−^, leading to the breakdown of the protective oxide layer and accelerating the transformation from the tetragonal to monoclinic phase [29,30,31,32,33].

The ICP analysis highlights this issue, revealing that yttrium release is highly dependent on the pH environment, with significant leaching observed during the acidic phases (pH 2) in both the constant immersion and pH cycling groups. The pronounced yttrium release during the acid-to-base cycling phase (Figure 1b) underscores how cyclic exposure to low pH exacerbates yttrium loss, consistent with the susceptibility of YSZ to chemical degradation under acidic conditions. In contrast, yttrium release is minimal at neutral (pH 7) and basic (pH 10) conditions, indicating that these environments help stabilize the material. Zirconium release, however, remains consistently low (below detectable levels), suggesting that the bulk zirconia matrix retains its structural integrity even in harsh acidic conditions, with degradation primarily affecting yttrium stabilization.

The XPS analysis further supports these findings, particularly the significant binding energy shift observed in the Zr 3d patterns (Figure 2a), indicating a transition from the tetragonal to the monoclinic phase in the zirconia. Tetragonal zirconia typically exhibits Zr 3d peaks at higher binding energies, while monoclinic zirconia is characterized by a shift toward lower binding energies due to changes in the electronic environment surrounding the Zr atoms [34,35,36]. The intensity increase and peak shift in the pH 2-immersed sample align with the well-established link between yttrium depletion and phase transformation. As yttrium ions leach out of the zirconia matrix, the tetragonal phase becomes unstable, promoting the formation of the monoclinic phase, a known consequence of LTD. This transformation is further evidenced by the absence of significant changes in the Y 3d patterns (Figure 2b), indicating that yttrium leaching occurs predominantly at the surface and grain boundaries without drastically affecting the bulk yttrium content until advanced stages of degradation.

The XRD results complement the XPS findings, showing a clear emergence of the monoclinic phase in not only the pH 2-immersed sample, as indicated by the (111) monoclinic peak at approximately 28.2° (2θ), but across all the samples immersed in all conditions. The presence of this peak is a well-known marker of phase degradation in YSZ. The tetragonal-to-monoclinic phase transformation leads to volumetric expansion and the formation of surface microcracks, compromising the material’s structural integrity. The increased intensity of the monoclinic peak in the solution-immersed sample, compared to the reference, highlights the accelerated phase transformation in the aquatic environment. Peak broadening in the XRD patterns also suggests decreased crystallinity and increased internal strain, both indicative of LTD.

To further elucidate the degradation mechanism of YSZ under acidic conditions, we closely examined the correlation between yttrium ion release and phase transformation behavior. Notably, during acid immersion and acid phases of the pH cycling tests, a sharp increase in yttrium concentration was observed, reaching peak values of 12–14 ppb. This leaching was temporally aligned with the emergence of the monoclinic (111) peak in the XRD pattern, particularly at 2θ ≈ 28.2°, suggesting that Y^3+^ loss directly compromises the tetragonal phase stability. Concurrently, XPS analysis of the acid-immersed sample revealed a −0.3 eV shift in the Zr 3d_5_/_2_ binding energy compared to the reference, indicative of altered electronic environments consistent with the monoclinic phase. Taken together, these findings support a mechanistic model in which surface protonation in acidic conditions disrupts Y–O bonds at grain boundaries, depleting yttrium and initiating a stress-driven tetragonal-to-monoclinic transformation. This transformation contributes to microcracking and surface degradation, which are characteristic features of low-temperature degradation (LTD) and can severely impact long-term mechanical reliability in clinical applications.

This combined evidence from ICP, XPS, and XRD analyses provides a comprehensive picture of how yttrium ion release in acidic conditions drives the phase transformation from tetragonal to monoclinic zirconia, ultimately threatening the long-term durability of YSZ as a dental implant material. Further studies, particularly mechanical testing, are necessary to quantify the impact of these phase transformations on the structural integrity of YSZ under prolonged acidic exposure.

## 5. Conclusions

This study demonstrates that the acidic environment of the oral cavity can significantly affect the longevity of YSZ used in dental implants. The pH cycling tests revealed that yttrium ion (Y^3+^) leaching is more pronounced under acidic conditions (pH 2), while zirconium ion (Zr^4+^) release remains consistently low across all pH environments. ICP analysis confirmed that the release of yttrium ions is strongly influenced by the acidity of the environment, with the highest concentrations observed during acid-to-base cycling phases. XPS further indicated that yttrium ion depletion destabilizes the tetragonal phase, as evidenced by a shift in the zirconium 3d binding energy. XRD results corroborated this finding, showing the emergence of the monoclinic phase in the acid-immersed samples, a process known to cause surface microcracking and mechanical degradation. These findings underscore the importance of understanding the chemical and structural changes in YSZ under acidic conditions, as yttrium leaching may compromise the long-term durability of dental implants.

## Data Availability

The original contributions presented in this study are included in the article. Further inquiries can be directed to the corresponding author.
